# Systemic inhibition of tumour angiogenesis by endothelial cell-based gene therapy

**DOI:** 10.1038/sj.bjc.6603883

**Published:** 2007-07-24

**Authors:** A Z Dudek, V Bodempudi, B W Welsh, P Jasinski, R J Griffin, L Milbauer, R P Hebbel

**Affiliations:** 1 Division of Hematology, Oncology and Transplantation, Department of Medicine, University of Minnesota, Minneapolis, MN, USA; 2 Department of Therapeutic Radiology, University of Minnesota, Minneapolis, MN, USA

**Keywords:** angiogenesis, postnatal vasculogenesis, tumour, blood outgrowth endothelial cell, gene therapy, endostatin

## Abstract

Angiogenesis and post-natal vasculogenesis are two processes involved in the formation of new vessels, and both are essential for tumour growth and metastases. We isolated endothelial cells from human blood mononuclear cells by selective culture. These blood outgrowth cells expressed endothelial cell markers and responded correctly to functional assays. To evaluate the potential of blood outgrowth endothelial cells (BOECs) to construct functional vessels *in vivo*, NOD-SCID mice were implanted with Lewis lung carcinoma cells subcutaneously (s.c.). Blood outgrowth endothelial cells were then injected through the tail vein. Initial distribution of these cells occurred throughout the lung, liver, spleen, and tumour vessels, but they were only found in the spleen, liver, and tumour tissue 48 h after injection. By day 24, they were mainly found in the tumour vasculature. Tumour vessel counts were also increased in mice receiving BOEC injections as compared to saline injections. We engineered BOECs to deliver an angiogenic inhibitor directly to tumour endothelium by transducing them with the gene for human endostatin. These cells maintained an endothelial phenotype and decreased tumour vascularisation and tumour volume in mice. We conclude that BOECs have the potential for tumour-specific delivery of cancer gene therapy.

Angiogenesis and vasculogenesis are two distinct processes involved in the formation of the vascular endothelial system that occur during embryogenesis and a variety of post-natal events, such as maturation of the corpus luteum, wound healing, proliferative retinopathy, rheumatoid arthritis, psoriasis, and cancer. During angiogenesis new vessels sprout from established vasculature. Angiogenic signals trigger complex changes in endothelial cells and extracellular matrix that result in remodelling, migration, and proliferation of pre-existing endothelium. The process begins with the proliferation of proximal endothelial cells that form capillary branches, with vessel lumen forming through anastomotic connections between capillary tips (Mustonen and Alitalo, 1995; Risau, 1997). Angiogenesis also depends on adhesive interactions between vascular cells of which multiple adhesion molecules are involved, such as *α*v*β*3, *α*v*β*5, *α*5*β*1, and *α*2*β*1 integrins; PECAM-1; and VE-cadherin (Heimark *et al*, 1990; Lampugnani *et al*, 1991, 1992; Brooks, 1996).

Vasculogenesis is a process whereby new vessels are assembled from coalescing and clustering endothelial progenitor cells (i.e., angioblasts). Both vasculogenesis and embryonic haematopoiesis are active in the mesoderm, and both involve descendent cells that differentiate from a common precursor cell, the haemangioblast (Coffin and Poole, 1988; Pardanaud *et al*, 1989; Choi *et al*, 1998). Recent studies have found intriguing evidence that vasculogenesis might play a postnatal role in neovascularisation (Crosby *et al*, 2000; Shintani *et al*, 2001). It has been reported that marrow-derived endothelial progenitor cells contribute to adult vasculogenesis (Tamura *et al*, 2004) and tumour angiogenesis (Rafii *et al*, 2002; Peters *et al*, 2005) by circulating through the vascular system and incorporating into the wall of newly formed vessels (Marchetti *et al*, 2002; Rafii and Lyden, 2003). Evidence that vasculogenesis is involved in neovascularisation has been found in studies on tumour vessels (Reyes *et al*, 2002), experimental retinopathy (Grant *et al*, 2002; Tomita *et al*, 2004; Butler *et al*, 2005), myocardial ischaemia (Kocher *et al*, 2001, 2006; Botta *et al*, 2004), wound healing (Asahara *et al*, 1999; Crisa *et al*, 1999), and hindlimb ischaemia (Takahashi *et al*, 1999; Kalka *et al*, 2000; Iwaguro *et al*, 2002). An important question that remains unanswered by the literature is whether circulating endothelial progenitor cells *per se* or their differentiated progeny are incorporated into the vascular wall (Moore *et al*, 2001).

Advances in our understanding of tumour vasculogenesis and stem cell development have allowed investigators to use endothelial-lineage cells as a potential tool for delivering genes to tumours. Endostatin is a potent inhibitor of angiogenesis and has demonstrated anti-tumour effects when delivered continuously (O'Reilly *et al*, 1997; Szary and Szala, 2001). Endostatin corresponds to the 20 kDa fragment derived from the COOH-terminal NC1 domain of type XVIII collagen (Oh *et al*, 1994; Rehn *et al*, 1994; O'Reilly *et al*, 1997). Endostatin disrupts the angiogenic process in many ways: it interferes with fibroblast growth factor-2 (FGF-2)-induced signal transduction, blocks endothelial cell motility, induces apoptosis, leads to G_1_ arrest of endothelial cells through inhibition of cyclin D1, blocks vascular endothelial growth factor-mediated signalling via direct interaction with its receptor tyrosine kinase (VEGFR-2, KDR/Flk-1) in human umbilical vein endothelial cells (HUVECs), and blocks tumour necrosis factor-induced activation of c-Jun NH_2_-terminal kinase and c-Jun NH_2_-terminal kinase-dependent proangiogenic gene expression. In addition, endostatin contains a heparin-binding motif, and may exert some of its antiangiogenic effects through interactions with the heparan sulphate proteoglycans, glypican-1, and -4, and binding to *α*5*β*_1_ integrin on the cell surface (Dhanabal *et al*, 1999; Shichiri and Hirata, 2001; Dixelius *et al*, 2002; Hanai *et al*, 2002; Kim *et al*, 2002; Yin *et al*, 2002; Abdollahi *et al*, 2004). Recombinant endostatin efficiently blocks angiogenesis and suppresses primary tumour growth and metastasis in experimental animal models without any apparent side effects, toxicity, or development of drug resistance (Boehm *et al*, 1997; O'Reilly *et al*, 1997; Marneros and Olsen, 2001). Use of endostatin in clinical trials in cancer therapy has been hampered by difficulties in protein production in large quantities, loss of biologic activity during long-term storage, and cumbersome daily administration requirements. Three phase I clinical trials and a recently reported phase II clinical trial of endostatin in patients with advanced neuroendocrine tumours have not demonstrated anti-tumour activity (Eder *et al*, 2002; Herbst *et al*, 2002; Thomas *et al*, 2003; Kulke *et al*, 2006). In the phase II study, steady-state trough levels were below the target therapeutic range.

We hypothesised that circulating endothelial cells could serve as a vehicle for continuous delivery of endostatin to tumour tissue. Using a murine model, we tested the ability of blood outgrowth endothelial cells (BOECs) cultured *ex vivo* from human peripheral blood mononuclear cells to target tumour vasculature. After establishing that BOECs home to tumour vessels, we evaluated the inhibitory effect of endostatin-transduced BOECs (EBOECs) on tumour growth and angiogenesis.

## MATERIALS AND METHODS

### Cell cultures

Healthy human volunteers donated 100 ml of venous blood after signing consent forms that had been approved by the University of Minnesota Institutional Review Board. Buffy coat mononuclear cells were prepared from diluted blood using Histopaque-1077 (Sigma Chemical, St Louis, MO, USA), as described previously. The cells were suspended in EBM-2/EGM-2 culture medium (Clonetics, San Diego, CA, USA) and plated into a culture well-coated with collagen I (Sigma Chemical). After 24 h, non-adherent cells and debris were removed by washing with medium. Thereafter, culture medium was changed daily until first passage, corresponding to expansion of approximately 3 × 10^4^ cells, and it was then changed every other day. After BOECs had expanded to approximately 10^7^ cells, they were lifted with trypsin (Gibco BRL, Grand Island, NY, USA) and plated onto a 10-cm culture dish coated with 6 *μ*g cm^−2^ type 1 collagen and 5 ml 1% gelatin containing 50 *μ*g ml^−1^ fibronectin (Sigma Chemical). Blood outgrowth endothelial cells were maintained in EGM-2 with 10% fetal bovine serum (FBS) and growth hormones as described previously (Lin *et al*, 2002). HUVECs were grown in M199 supplemented with Endo-Gro (VEC Technologies, Rensselaer, NY, USA), heparin, MEM sodium pyruvate and FBS. Lewis lung carcinoma cells were maintained in RPMI with 10% FBS. PT67 cells (Clontech Laboratories, CA, USA) were maintained in DMEM with 10% FBS. All cell lines were maintained at 37°C in a 5% CO_2_ humidified atmosphere.

### BOEC tracking in tumour-burdened mice

All animal studies were approved by University of Minnesota Institutional Animal Care and Use Committee and were in compliance with the UKCCCR Guidelines for the welfare of animals in experimental research. Mice were kept in pathogen-free conditions. FSaII carcinoma cells were implanted s.c. in Nu/Nu, Balb/C Nu mice. A total of 36 mice were divided into nine experimental groups (*n*=4 in each group): control animals with no radioactive chromium (one group), chromium alone (four groups) and animals injected with chromium-labelled BOEC (four groups killed at 1, 4, 48, and 72 h after injection). Once palpable tumours appeared, mice were infused through the tail vein with 250 000 BOECs labelled with chromium 51. Tumours and other organs were removed at 1, 4, 48, and 72 h after BOEC injection, and radioactivity was measured with a well-type gamma counter (1282 Compugamma; Pharmacia LKB Wallac, Turku, Finland) and calculated per gram of tissue.

Twenty-nine NOD/SCID mice bearing Lewis lung carcinoma tumour were injected through the tail vein with 250 000 BOECs, BOECs or GBOECs (BOEC transduced with empty vector). The presence of BOECs, GBOEC or EBOECs in tumour tissue was detected by quantitative real-time PCR for human *β*2 microglobulin (hB2M) at the time of killing of mice (16–21 days after endothelial cells injection) (Table 1). Detection of hB2M in murine tumour tissue was performed by isolating the total genomic DNA from murine tissues and tumours using DNeasy Tissue Kit (Qiagen, Valencia, CA, USA) and diluting it to 100 ng *μ*l^−1^ with PCR grade water. Dual-colour genomic DNA real-time quantitative PCR was performed to quantify the amount of human BOEC genomic DNA existing in each murine organ and tumour by using ICycler (Bio-Rad Laboratories CA, USA). The primers and 3′-labelled fluorescence FAM-probe for hB2M were designed to amplify human genomic DNA specifically. The sequences are as follows: hB2M sense primer-5′-GCTGGATTGGTATCTGAGGCTAG-3′; hB2M antisense primer-5′-GCTGTTCCTACCCATGAATACAT-3′; hB2M probe-5′-AAGGGCTTGTTCCTGCTGGGTAGCTCTAAAC-FAM-3′. Besides hB2M primers and probe, the universal GAPDH primers and 3′-labelled fluorescence VIC-probe (TaqMan Rodent GAPDH Control Reagents from Applied Systems; Weiterstadt, Germany) were included in each PCR to amplify both human and murine DNA for internal control. The standard curve was set up by serially diluting human BOEC genomic DNA in murine tissue genomic DNA in several different rations. Each dual-colour real-time PCR contained 500 ng of total genomic DNA from murine tissue, 200 nM of each primer, 150 nM of each probe and iQ supermix (Bio-Rad Laboratories). The amplification condition consisted of denaturing DNA and activating polymerase at 95°C for 5 min followed by 60 cycles of two-step PCR at 95°C for 30 s and 60°C for 30 s. The modified threshold cycle value (*C*_t_) of each sample (*C*_t_ of hB2M divided by *C*_t_ of GAPDH) was used to calculate the percentage of human cells in murine tissue based on the standard curve.

BOEC cells were labelled with carboxyfluorescein succinimidyl ester (CFSE) as described previously (Lyons and Parish, 1994). A total of 10^6^–10^7^ cells were washed twice with phosphate-buffered saline (PBS) and then incubated with 5 nM CFSE in PBS for 3 min at room temperature. The cells were washed two times with RPMI medium supplemented with 10% FBS.

### Construction of endostatin expression vector

**Figure 1:**
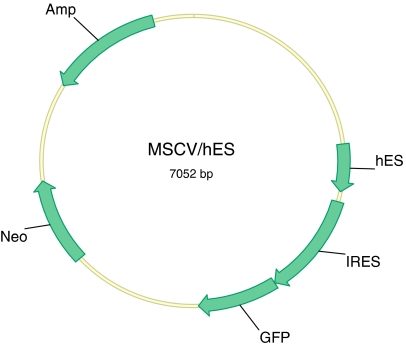
Retroviral vector expressing human endostatin. hES: human endostatin, IRES: internal ribosome entry site for transcription of single biscistronic mRNA transcript of hES and fluorescence gene, GFP: green fluorescent protein, Neo: Neomycin resistance gene for selection in mammalian cells, Amp: Ampicillin resistance gene for selection in bacterial cells.

The 660-bp DNA fragment containing full-length hES (human endostatin) cDNA and BM40 (human extracellular matrix protein) signal peptide (a gift from Dr Bjorn R Olsen and Dr Naomi Fukai, Department of Cell Biology, Harvard Medical School) was cloned into an intermediate vector, pTracer-CMV2 (Invitrogen Corp., CA, USA), and named pTracer/hES. The hES cDNA was then subcloned in between the *Bgl*II and *Hpa*I cloning sites of MIRG and named MIGR/hES. The MIGR/hES contained BM40 signal peptide and hES cDNA, followed by internal ribosome re-entrance site (IRES) and green fluorescent protein (GFP) cDNA (Figure 1).

### Construction of retrovirus expressing endostatin

MIGR/hES DNA and pcDNA3.1(−) plasmid DNA in a 10 : 1 ratio were co-transfected into a virus-producing cell line, Retro Pack PT67 (Clontech Laboratories), using Fugene6 (Roche Molecular Biochemical, IN, USA) according to the protocol supplied by the company. The stable cell line containing MIGR/hES (named PT67/hES) was then established by Neomycin selection for 2 to 3 weeks. Conditioned medium from PT67/hES was collected 48 h later and filtered through a 0.45-*μ*m cellulose acetate filter and stored at −70°C. Similarly, virus containing only GFP was produced to use as a control.

### Gene transfer to blood outgrowth endothelial cells

Blood outgrowth endothelial cells at 70% confluence were transduced with retroviral particles. GBOECs were transduced with an empty vector expressing GFP. EBOECs were transduced with a vector expressing both GFP and human endostatin. Serial transductions were performed to gradually enrich positive cells that were collected by fluorescence-activated cell sorting (FACS).

### Quantifying expression of human endostatin

Levels of human endostatin were measured in cultured supernatants from EBOEC using the ELISA kit (R&D Systems, Minneapolis, MN, USA). All samples were measured in triplicates.

### Cell proliferation assay

After 24 h, conditioned media were collected from flasks with HUVEC, BOEC, and EBOEC cultures. Each medium was filtered through 0.45 *μ*m filter unit, and aliquots stored at −20°C for further use. HUVEC were seeded in 96-well plates (precoated with 1% gelatin) at a density of 2 × 10^3^ cells well^−1^ in M199 medium with 5% FBS. Thawed conditioned medium supplemented with 50 ng ml^−1^ of human basic fibroblast growth factor (bFGF) was added to the wells in nine replicates per treatment group. Positive control wells had HUVEC conditioned medium supplemented with 50 ng ml^−1^ of bFGF. HUVEC proliferation was determined at 72 h by MTT (3–4,5-dimethylthiazol-2,5-diphenyl tetrazolium bromide) assay (Roche Diagnostic GmbH, Penzberg, Germany) according to the manufacturer's protocol. The number of viable cells was quantified spectrophotometrically at 575 nm using an ELISA microplate reader.

### Tube formation assay

HUVECs (1 × 10^5^ cells) were plated on wells precoated with 200 *μ*l of Matrigel (10 mg ml^−1^, Becton Dickinson and Company, Franklin Lakes, NJ, USA). Cultured supernatant from BOEC or EBOEC was added to each well and incubated for 18 h. Tube formation was evaluated under inverted microscope (Olympus CK30-F100, Japan).

### Evaluating effects of GBOEC and EBOEC on tumour vessel density and growth

Lewis lung cells were injected s.c. into 29 immunocompromised NOD/SCID mice. Seven days after tumour implantation, mice were injected through the tail vein every other day for a total of three times with 2 × 106 GBOECs, EBOECs or saline (8 mice group^−1^). Five mice were also injected with BOEC. Twenty-four hours before the first injection, 200 *μ*l of anti-asialo GM1 (Wako Chemicals, Richmond, VA, USA) antibody, which had been reconstituted in PBS, was injected intravenously (i.v.) in the lateral tail vein of each mouse; this procedure was performed to eliminate murine NK cells and increase the likelihood of human BOEC survival in the murine host. Tumour size was determined by caliper measurements, and tumour volume *V* was calculated using the following formula: *V*=(0.536 × length × width × width).

Animals were killed at day 24 after BOEC injection. Tumour tissue was stained with an antibody recognising CD31 antigen (Accurate Chemical and Scientific Corp., Westbury, NY, USA), which is present on murine and human endothelial cells. Images from slides were analysed with Metamorph image analysis software and stored as TIFF files. Files were then opened in Adobe Photoshop (Adobe Inc., Mountain View, CA, USA). The greyscale images were then adjusted to 256 scales of grey using the auto-contrast function. One pixel depth Gaussian blur was used to smooth edges. After threshold adjustment, images were reduced to black and white pixels. All microvessels were reduced to black lines (Photoshop Adobe processing toolkit (RGI) command: Filter: Erode and Skeletonize). Using Adobe Image processing toolkit (Adobe command: Filter: IP^*^ lines+points, total length), we were able to calculate total vessel length. A final estimation of the total vessel count was obtained using the formula: vessel number=(vessel ends+vessel branch points)/2.

### Immunohistochemistry

The phenotype of EBOEC and GBOEC were determined by morphology, expression of endothelial markers (flk-1, VE-cadherin, and CD31) and uptake of acetylated low-density lipoprotein (LDL). Uptake of acetylated LDL is one of the functional methods of identification of endothelial cells (Voyta *et al*, 1984). Expression of markers for monocytes (CD14) and leukocyte common antigen (CD45) were used as negative controls. EBOECs were also evaluated for presence of endostatin by fluorescence microscopy.

Cells were seeded in Lab-tek 4-well chamber slides (Nalge Nunc, Naperville, IL, USA) at approximately 80% confluency. Cells were washed three times with PBS, fixed in 4% paraformaldehyde for 10 min and permeabilised with Triton X-100 for 1 min. Cells were then blocked in PBS supplemented with 3% bovine serum albumin (BSA) (Sigma, St Louis, MO, USA) for 1 h at 37°C. Subsequently, cells were incubated for 1 h at 37°C with primary antibodies: endostatin, flk-1, VE-cadherin, CD14, CD45 (Santa Cruz Biotechnology, Santa Cruz, CA, USA), and CD31 (BD Biosciences, Bedford, MA, USA). Cells were then washed five times with PBS and incubated for 1 h at 37°C with anti-rabbit, anti-goat, and anti-mouse secondary antibodies conjugated with TRITC (Jackson Immuno Research, West Grove, PA, USA) and diluted in blocking solution. Cells were then washed five times with PBS and incubated with DAPI (Molecular Probes, Eugene, OR, USA) diluted in PBS for 10 min at room temperature. Finally, cells were washed five times with PBS, and pictures were taken with the Nikon Eclipse TE200 fluorescent microscope (Nikon, Tokyo, Japan) using filters for TRITC, DAPI, and GFP.

To identify human BOEC or EBOEC in tumour tissue, paraffin-embedded murine tumour was cut into 5-*μ*m sections and deparaffinised before treatment with 0.1% trypsin to unmask antigens. Sections were blocked with 1% BSA and 0.2% Tween-20 in PBS for 30 min or with an avidin/biotin blocking kit (Vector, Burlingame, CA, USA). Primary antibody recognising *β*2-macroglobulin, CD31 (Accurate Chemical and Scientific corp.), or polyclonal goat anti-human endostatin (R&D Systems) was used for 1 h at room temperature. Secondary anti-rabbit or anti-goat biotinylated antibodies (Jackson ImmunoResearch Labs) were applied respectively for 30 min. Avidin/biotin enzyme complex (Vector) was used for signal amplification, followed by DAB peroxidase substrate kit (Vector). In controls primary antibodies were omitted. Nuclei were counterstained with haematoxylin (Vector) and photographed at × 900 magnification. For immunofluorescent staining of *β*2-macroglobulin and human endostatin in tumour sections, we used donkey anti-rabbit TRITC-labelled and anti-goat FITC-labelled secondary antibodies (Jackson ImmunoResearch labs). 4′,6-diamidino-2-phenylindole, a blue fluorescence stain that binds to nucleic acid, was used as a nuclear counterstain (Invitrogen).

For microvessel density CD31-stained tumour sections were scanned at low power, and the areas of greatest CD31-positive density were chosen for quantification of intratumoural vessel density. Microvessel density counts were determined by two blinded observers.

### Detection of human endostatin in murine tumour

For Western blot detection of human endostatin, we extracted tumours from *in vivo* studies and placed them in 1 ml of ice-cold lysis buffer (20 mM Tris–HCl (pH 7.5), 150 mM NaCl, 1 mM Na_2_EDTA, 1 mM EGTA, 1% Triton, 2.5 mM sodium pyrophosphate, 1 mM *β*-glycerophosphate, 1 mM Na_3_VO_4_, 1 *μ*g ml^−1^ leupeptin, 1 mM PMSF) (Cell Signalling, Boston, MA, USA). Lysed cells were immediately sonicated on ice at intermediate settings five times for 15 s each and centrifuged at 10 000 r.p.m. for 30 min at 4°C. Protein concentration was determined by BioRad protein assay (BioRad Laboratories, Hercules, CA, USA), and samples were prepared for SDS–PAGE, followed by electroblotting onto PVDF membrane. The blot was blocked 1 h in Tris-buffered saline (BioRad Laboratories) containing 0.05% Tween-20 and 5% BSA (Sigma-Aldrich, St Louis, MO, USA) at room temperature and incubated overnight at 4°C with appropriate amounts of endostatin antibody (Abcam, Cambridge, MA, USA) and actin antibody (Santa Cruz Biotechnology). Immunoreactive protein was detected by incubating blots with AP-conjugated secondary antibody and ECF fluorescent substrate, which was then visualised by a Storm™ fluorescent scanning system.

### Statistical analysis

A *t*-test was used to compare the experimental and control groups. A nonparametric Mann–Whitney rank sum test was used for non-normal distributions. For tumour volumes log volume data were analysed with one-way ANOVA for parametric variables and assessed by Turkey's method using Instat Software (GraphPad Software, San Diego, CA, USA).

## RESULTS

### BOECs accumulate in tumour tissue and enhance tumour vessel growth

**Figure 2:**
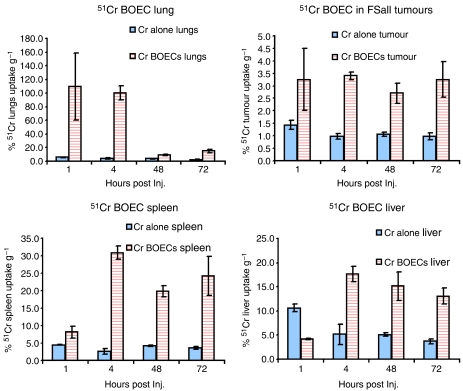
BOEC tracking in tumour-burdened mice. FSaII carcinoma cells were implanted s.c. in 36 mice. Once palpable tumours appeared, mice were infused through the tail vein with chromium 51 (blue bars), or BOECs labelled with chromium 51 (red bars). One control group had only saline injection. Tumours and other organs were removed at 1, 4, 48, and 72 h after BOEC injection (*n*=4), and radioactivity was measured with a gamma counter to track the distribution of BOECs.

The systemic distribution of radiolabeled BOECs injected through the tail vein of Nu/Nu, Balb/C Nu animals bearing s.c. implanted FSaII carcinoma cells tumours varied over time. One hour after BOEC administration, accumulation of BOECs was higher in lung and tumour tissue as compared to the liver, kidney, and spleen. At 72 h post-injection, BOEC concentration remained the same in the spleen, liver, and tumour tissue, but decreased in lung (Figure 2).

**Figure 3:**
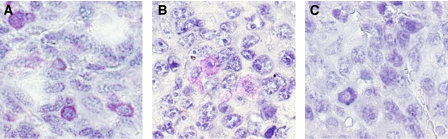
BOECs and EBOECs in tumour tissue. (**A**) Tumour sections from animals injected with BOECs were stained with anti-human *β*2-macroglobulin. (**B**) Tumours from animals injected with EBOECs were stained with antibody recognising endostatin. (**C**) The primary antibody was omitted as a control. Original magnification × 900. Micrographs are representative of tumour sections stained from BOEC-injected (*n*=5) and EBOEC-injected (*n*=8) mice.

**Figure 4:**
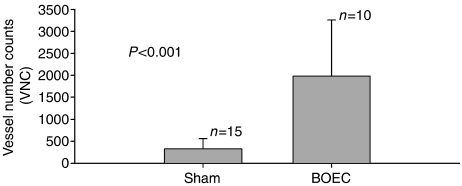
BOECs increase blood vessel density in tumour. Mice with Lewis lung carcinoma tumours were injected with saline or BOEC and killed 24 days later. Tumour tissue was stained with an antibody recognising CD31, which is present on murine and human endothelial cells. Images from slides were analysed with a Metamorph image analysis programme and processed with Adobe Photoshop. Wild-type BOECs greatly enhanced the number of vessels found in tumours (*P*<0.0001).

Immunostaining of tumour tissue samples (from five animals with implanted Lewis lung cancer and injected via tail vein with BOEC, and from eight animals with implanted Lewis lung cancer injected with saline) with *β*2-macroglobulin confirmed that BOECs were present in tumour (Figure 3A) in BOEC-injected animals only. In addition, BOECs were detectable 21 days after injection (2–3 BOECs/100 000 tumour cells) using real-time PCR for detecting hB2M as a BOEC marker (Table 1). CFSE-labelled BOECs were detected in tumour upto 10 days after injection (data not shown). Vessel count calculated from CD31-stained tumour slides was over four times higher in tumours from BOEC-injected mice compared to mice injected with DPBS solution) (Figure 4).

### EBOECs display normal endothelial cell phenotype

**Figure 5:**
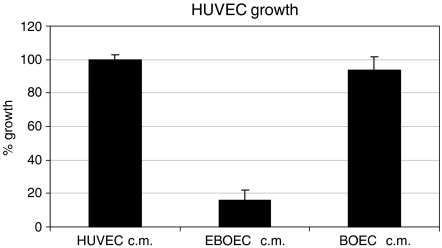
EBOEC-conditioned medium inhibits growth of HUVECs. The proliferation of HUVECs cultured in conditioned medium from HUVECs, BOECs or EBOECs was assessed by MTT assay. Experiments were performed in nine replicates per treatment group. Results of Student's *t*-test showed a significant reduction in the number of HUVECs cultured in EBOEC-conditioned medium compared to the number of HUVECs cultured in HUVEC- or BOEC-conditioned medium (*P*<0.001).

EBOECs and GBOECs were successfully produced by retroviral gene transfer by sequential transduction and sorting by FACS. The presence of transgene in EBOECs was confirmed by PCR. Immunostaining of tumour tissue samples with an antibody recognising human endostatin confirmed that EBOECs were present in tumour (Figure 3B). EBOECs produced 1.35 ng ml^−1^ of endostatin (supernatant from 2 × 10^5^ cells) in 24 h. Figure 5 shows HUVEC growth inhibition by EBOEC-conditioned medium (*P*=0.001). In addition, EBOEC-conditioned medium inhibited tube formation by HUVECs, whereas BOEC-conditioned medium had no such effect (data not shown).

**Figure 6:**
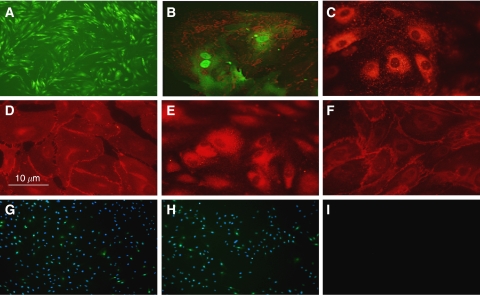
Phenotype of EBOECs. Morphology of EBOECs (**A**). EBOEC express endostatin (red)(**B**), VWF (**C**), VE-cadherin (**D**), Flk-1 (**E**), CD31 (**F**), but do not express CD14 (**G**), nor CD45 (**H**). Negative control (**I**). DAPI was filtered from positive stains (**B**–**F**) due to GFP interference. Images are representative of triplicate chamber slides.

**Figure 7:**
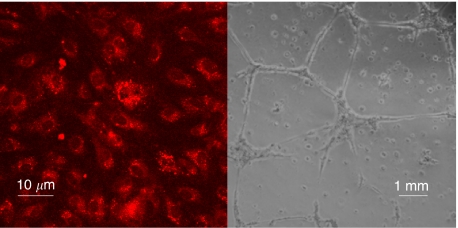
EBOECs maintain endothelial cell function. Uptake of acetylated LDL is a method for the identification of functional endothelial cells. EBOECs take up acetylated LDL (left) and form vascular tubes in culture (right). Images are representative of triplicate slides (LDL uptake) or wells (vascular tubes).

Phenotypic characterisation showed that EBOECs and GBOECs retained the general phenotype of BOECs. A ‘cobblestone’ morphology characteristic of parental BOECs was observed using light microscopy (Figure 6A). These cells were positive for expression of endothelial cell markers vWF, VE cadherin, flk-1, CD31 (PECAM), but not monocytes or lymphocytes markers CD14 or CD45 (Figures 6C–H). EBOECs also expressed endostatin (Figure 6B). A normal phenotype for EBOECs and GBOECs was further confirmed by the formation of angiogenesis-like vessel networks (albeit less robust for EBOECs than GBOECs) and uptake of acetylated-LDL (Figure 7). Acetylated-LDL uptake is one of the hallmarks of endothelial cell physiology (Voyta *et al*, 1984).

### EBOECs inhibit tumour growth

**Figure 8:**
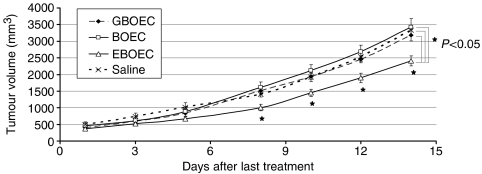
Therapeutic effect of EBOECs on tumour size. Lewis lung carcinoma cells were injected s.c. into eight immunocompromised NOD/SCID mice per group, with the exception of the BOEC group, which had five mice. Three injections of anti-asialo GM1 antibody were used to eliminate murine NK cells and increase the likelihood of human BOEC survival in the murine host. Seven days after tumour implantation, mice were injected through the tail vein every other day for a total of three times with 2 × 106 GBOECs, EBOECs, BOECs in saline suspension or equivalent volume of saline. Significant differences in tumour volumes for treatment with EBOEC *vs* three control groups were seen from day 8 through day 14 after last treatment (^*^P<0.05).

**Figure 9:**
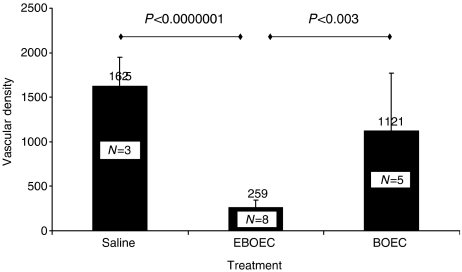
EBOECs decrease blood vessel density in tumour. Tumour sections from mice injected with BOEC (*n*=5), EBOEC (*n*=8) or saline (*n*=3) were stained with an antibody against CD 31. EBOEC-injected mice had tumours with fewer blood vessels than BOEC- or saline-injected mice (*P*<0.003 and *P*<0.0000001, respectively).

**Figure 10:**
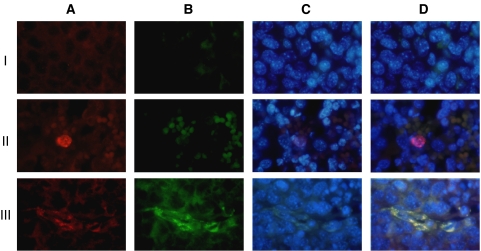
EBOECs in tumour tissue. Tumour samples were collected from the 24 mice injected i.v. with Lewis lung carcinoma cells. Column A: cells stained for the presence of human *β*2-macroglobulin. Column B: cells stained for the presence of human endostatin. Column C: cells stained with the nuclear stain DAPI. Merged images of all three stainings are shown in column D. Row I: tumour from saline-injected animals (*n*=8). Row II: tumour from GBOEC-injected animals (*n*=8). Row III: tumour from EBOEC-injected animals (*n*=8). Human BOEC (GBOEC or EBOEC) in tumour tissue can be seen in panels A-II, A-III, D-II, and D-III. Endostatin-producing BOEC (EBOEC) can be found in EBOEC-injected animals (B-III and D-III). Images are representative from tumour tissue collected from all animals in *in vivo* experiment described in Figure 8.

**Figure 11:**
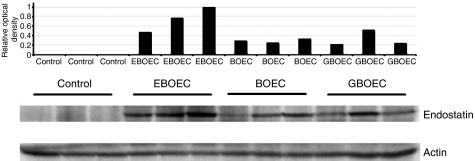
Expression of endostatin by EBOEC in tumours. Human endostatin was detected by Western blot in tumours in animals injected with EBOEC, although a small amount of endostatin was also seen in tumours in animals injected with BOEC and GBOEC (three mice per group). Endostatin was not detected in tumours from saline-injected mice (not shown).

Lewis lung cells injected s.c. into NOD/SCID mice formed palpable tumours at day 4 after implantation. Seven days after implantation, mice were injected either with BOECs, GBOECs, EBOECs or saline. Tumours in mice injected with EBOECs were significantly smaller than tumours injected with BOECs, GBOECs or saline (*P*<0.05 for each group compared with EBOEC) (Figure 8). Tumour sections were stained with an anti-CD31 antibody to evaluate tumour vascularity. A greater density of vessels was seen in tumours from BOEC-treated mice compared to EBOEC-treated mice (Figure 9). EBOECs and GBOECs were detected in tumour tissue from their respective animals (Figure 10). Human endostatin was detected by Western blot in tumours in mice injected with EBOECs, but not in tumours of mice injected with saline. A small amount of human endostatin was also detected in tumour tissue from BOEC- and GBOEC-injected animals (Figure 11).

## DISCUSSION

Our investigation establishes two key steps in the development of a therapeutic strategy for delivering tissue-specific gene therapy. First, we demonstrate that circulating BOECs target tumour tissue and augment vessel growth through incorporation into vessel endothelium. Results of real-time PCR and chromium-51 labelling of BOECs showed that these cells migrate to sites of active vascular growth, such as tumour, liver, and spleen (Figures 2 and 3). This observation is consistent with data reported by Jevremovic *et al*, who has also found that endothelial cell precursors migrate to the tumour vasculature. In addition, CD31-stained tumours from mice injected with BOECs showed an increase in tumour vasculature (Figures 4 and 9). Taken together, our results suggest that BOECs integrate into and stimulate tumour vasculature growth (Figure 4).

Second, we demonstrate that inhibition of tumour growth in EBOEC-injected mice was attributable to the inhibitory effect of endostatin on tumour angiogenesis. We observed a 28% reduction in tumour size in mice receiving EBOEC injections (Figure 8). In addition, the biological activity of the endostatin present in the supernatant of EBOECs was verified by its ability to inhibit specifically the proliferation and tube formation by HUVECs *in vitro* (Figure 5). Both *in vivo* and *in vitro* results demonstrate that BOECs transfected with retrovirus containing the endostatin gene are capable of long-term secretion of endostatin.

Recent studies report conflicting results with regard to the extent of endothelial cell integration into the tumour vasculature. While our findings demonstrate that BOECs integrate into tumour vessels, the integration occurs at a very low level. This finding is consistent with recent reports, which show that bone marrow-derived endothelial precursor cells migrate to tumour vasculature at similarly low levels (De Palma *et al*, 2003; Machein *et al*, 2003; Droetto *et al*, 2004; Larrivee *et al*, 2005). However, these results contrast with a previous report demonstrating that 90% of blood vessels in B6RV2 tumours are composed of bone marrow-derived endothelial cells (Lyden *et al*, 2001). These incongruous results might be explained by the existence of multiple factors directing endothelial cell migration, including initial tumour size, extent of vascularity, differences in detection time after cell injection, total number of injected cells, and differences in the microenvironment of marrow-derived cells *vs* BOECs *in vivo*. In addition, the composition of angiogenic factors varies widely between the microenvironment of a s.c. tumour model and orthotopic model (Yancopoulos *et al*, 2000). A low level of endothelial cell integration into the tumour vasculature may pose a potential problem for the delivery of sufficient quantities of gene product to sites of tumour angiogenesis. We are currently exploring, however, whether our observation was not solely due to the decreased incorporation of human endothelial cells into a murine host.

Our findings provide a rationale for developing antiangiogenic BOECs as an approach to gene therapy-mediated cancer treatment. Other gene therapies utilise viral or non-viral delivery systems, and the main drawback of these strategies is the absence of long-term expression of therapeutic proteins due to the probable immune response by the host to foreign material (Harrington *et al*, 2002). Early attempts to overcome these drawbacks involved delivery of genes to tumour sites via cell-based carriers. The use of T cells and macrophages has been extensively studied due to the homing properties of these immune cells. Recently, problems with sustained production of antiangiogenic proteins were overcome by adeno-associated virus-mediated intratumoural delivery (Ma *et al*, 2002b) or systemic delivery through intramuscular injection of angiostatin for treatment of intracranial tumours (Ma *et al*, 2002a) or endostatin for treatment of ovarian carcinoma (Subramanian *et al*, 2006). Long-term survival of mice with intracranial human glioblastoma was also seen when the Sleeping Beauty transposon system was used for the transfer of a gene encoding soluble vascular endothelial growth factor receptor or a fusion gene for angiostatin–endostatin (Ohlfest *et al*, 2005).

Our study demonstrates that BOECs can be engineered to produce antiangiogenic proteins *in vivo* on a continuous basis without the need for daily administration of recombinant protein. Two potential advantages of using BOECs as a delivery system in gene therapy include the ability to grow autologous BOECs from peripheral blood and the ease of manipulating them to express any gene of interest. A possible drawback of using BOEC for antiangiogenic gene therapy would be the potential for increased tumour vessel outgrowth and increased tumour growth, if silencing of anticancer genes in therapeutic BOECs were to occur *in vivo*.

Based on our findings with endostatin-transfected BOECs, we propose BOECs as an appropriate delivery vehicle for novel antiangiogenic proteins. This therapeutic strategy also has implications for the specific targeting of other classes of anticancer agents to the tumour microenvironment using autologous BOECs.

## Figures and Tables

**Table 1:** Quantitative real-time PCR detection of the BOEC biomarker hB2M

			**Human cells in tumour**		
**Type of cells injected**	**Tumour harvested: day after first injection**	**Tumour sample**	**%**	**Mean**	**s.d.**
BOEC	21-day	BOEC	0.00001	0.00271	0.00271
	21-day	BOEC	0.00542		
	21-day	BOEC	0.00271		
					
EBOEC	22-day	EBOEC	0.00147	0.00311	0.00137
	21-day	EBOEC	0.00376		
	22-day	EBOEC	0.00461		
	21-day	EBOEC	0.00261		
					
GBOEC	20-day	GBOEC	0.00257	0.00315	0.00103
	16-day	GBOEC	0.00434		
	20-day	GBOEC	0.00253		
					

Abbreviations: BOEC=blood outgrowth endothelial cells; EBOEC=endostatin-transduced BOECs.
